# Ionic Mechanism Underlying Optimal Stimuli for Neuronal Excitation: Role of *Na* ^+^ Channel Inactivation

**DOI:** 10.1371/journal.pone.0045983

**Published:** 2012-09-26

**Authors:** John R. Clay, Daniel B. Forger, David Paydarfar

**Affiliations:** 1 National Institute of Neurological Disorders and Stroke, National Institutes of Health, Bethesda, Maryland, United States of America; 2 Department of Mathematics and Center for Computational Medicine and Bioinformatics, University of Michigan, Ann Arbor, Michigan, United States of America; 3 Departments of Neurology and Physiology, University of Massachusetts Medical School, Worcester, Massachusetts, United States of America; 4 Wyss Institute for Biologically Inspired Engineering, Harvard University, Boston, Massachusetts, United States of America; Instituto de Neurociencias de Alicante UMH-CSIC, Spain

## Abstract

The ionic mechanism underlying optimal stimulus shapes that induce a neuron to fire an action potential, or spike, is relevant to understanding optimal information transmission and therapeutic stimulation in the nervous system. Here we analyze for the first time the ionic basis for stimulus optimality in the Hodgkin and Huxley model and for eliciting a spike in squid giant axons, the preparation for which the model was devised. The experimentally determined stimulus is a smoothly varying biphasic current waveform having a relatively long and shallow hyperpolarizing phase followed by a depolarizing phase of briefer duration. The hyperpolarizing phase removes a small degree of the resting level of *Na* ^+^ channel inactivation. This result together with the subsequent depolarizing phase provides a signal that is energetically more efficient for eliciting spikes than rectangular current pulses. Sodium channel inactivation is the *only* variable that is changed during the stimulus waveform, other than the membrane potential, *V*. The activation variables for *Na* ^+^ and *K* ^+^ channels are unchanged throughout the stimulus. This result demonstrates how an optimal stimulus waveform relates to ionic dynamics and may have implications for energy efficiency of neural excitation in many systems including the mammalian brain.

## Introduction

The features of neural stimulation that cause action potentials, or spikes, are relevant to a number of questions in neuroscience [1]. One aspect of this problem that has attracted considerable interest is the determination of the precise stimulus waveform shape or shapes that optimally elicit spikes in a neuron [1]–[6]. We recently addressed this question using squid giant axons and the Hodgkin and Huxley model of the action potential [1], [7]–[8]. Our approach utilizes a stochastic search methodology in which an array of stochastically determined stimulus shapes is considered, including those that displace the membrane from rest to firing threshold. When the overall intensity of the stimulus array is reduced to a level at which action potentials rarely occur, then such rarely occurring suprathreshold stimuli are candidate optimal shapes for eliciting an action potential. We had previously shown that this method yields optimal waveforms that are similar to those found analytically using variational techniques [2]. A key finding in our work is that the optimal stimulus shape is biphasic, consisting of a relatively long lasting hyperpolarizing phase followed by a briefer duration depolarizing phase [1]. This shape is remarkably similar to those found in other preparations in which noisy stimulus trains were administered and a preferred stimulus shape for eliciting a spike was determined using spike triggered averaging [9]–[12].

In the present study we investigate the ionic mechanism underlying the biphasic shape for stimulus optimality. The key factor in the analysis is sodium channel inactivation. The rest potential for many neurons lies close to the midpoint of the inactivation curve. Consequently, small changes of the membrane potential can produce significant changes in the inactivation variable. In particular, hyperpolarization of the membrane potential effectively increases the availability of sodium channels to be activated during the subsequent depolarizing phase of the biphasic stimulus. This result is related to anode break excitation, a well-known method for triggering an action potential [7], [13]. Our analysis provides a novel view of this mechanism.

## Methods

### Stochastic stimulation of squid giant axons

Our method for stimulating the squid giant axon preparation has been previously described [1], [14]. Stochastically varying current was administered to the axon for periods of 100 seconds using stimulus profiles generated by computer with a simple model of stochastically summated PSCs (postsynaptic currents). The analysis was carried out with MatLab (The MathWorks, Natick, MA). Excitatory and inhibitory PSCs were generated independently, each with a Poisson rate having a mean of 10 events per msec. Each PSC had an exponential rise time constant of 0.25 msec and a decay time constant of 1 msec [14]. Specifically, each PSC was given by ±(1−exp(−*l_1_t*))exp(−*l_2_t*) with the plus symbol referring to EPSCs, the minus symbol referring to IPSCs, *l_1_* = 4 msec^−1^ and *l_2_* = 1 msec^−1^. Each PSC is a discrete, smoothly varying waveform. The randomness, i.e., the “noise” of the signal, was determined by two factors: the times of occurrence of each PSC, and the polarity of each PSC. Both were determined by means of a random number generator. The input was determined from a summation of all PSCs. The random arrival of PSCs at the cell body was mimicked by this approach. The computed stimulus profiles were converted to an analog stimulus using a D-A converter (National Instruments, Austin, TX) controlled by software (LabView 6, National Instruments). The mean current for any run was zero because the excitatory and inhibitory PSCs had identical profiles and Poisson distributions.

### Simulations of Excitability

Simulations were carried out in Mathematica (Wolfram Research, Champaign, IL) using the Hodgkin and Huxley model of the action potential [7]–[8],
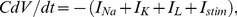
(1)where *C* is membrane capacitance (*C* = 1 µF/cm^2^), *V* is membrane potential in mV, *t* is time in msec, and *I_Na_*, *I_K_*, *I_L_*, and *I_stim_* are the *Na* ^+^, *K* ^+^, leak, and stimulus currents components, respectively, all in µA/cm^2^. The first three of these are
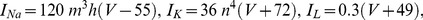
(2)where *m* and *n* are time dependent variables related to *Na* ^+^ and *K* ^+^ channel activation respectively (see below), and *h* is a time dependent variable that describes *Na* ^+^ channel inactivation. These variables are given by
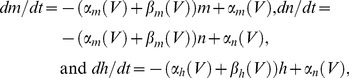
(3)with
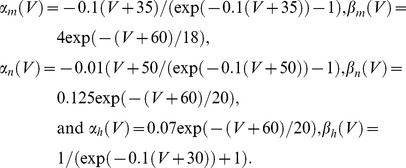
(4)


All *α*'s and *β*'s are in units of msec^−1^. The current stimulus term, *I_stim_*, is either a rectangular pulse or a mathematical function given below that we have used to describe the experimentally determined optimal stimulus from our recent work [1]. The Hodgkin and Huxley model can be considered to consist of four independent gating particles for each channel: three activation, or *m* gates, and an inactivation gate, or *h* gate, for *Na* ^+^ channels; and four activation, or *n* gates, for *K* ^+^ channels [15]. Any single gate can be considered to be in either a closed or open state. All four gates of any single channel must all be open for the channel itself to be open. Consequently, activation is described by *m* ^3^ for *Na* ^+^ channels and *n* ^4^ for *K* ^+^ channels [15].

## Results

### Optimal stimulus

**Figure 1:**
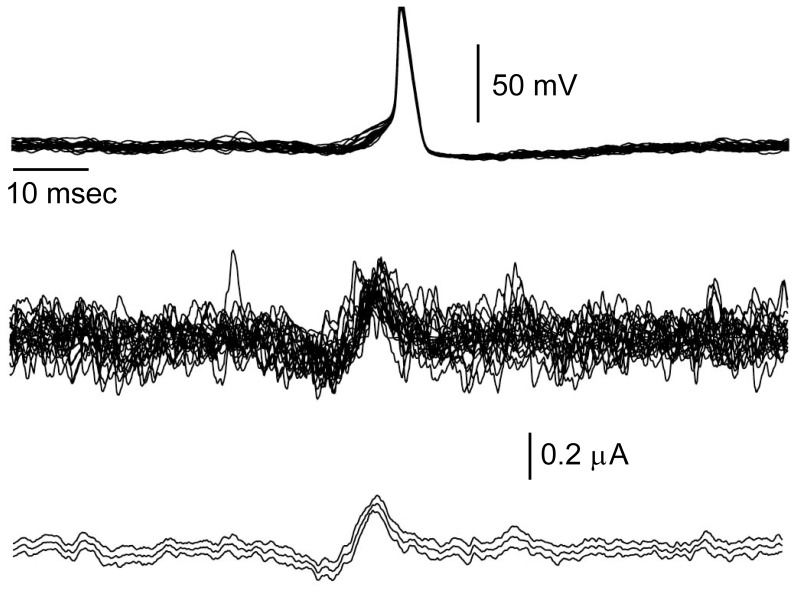
Spike triggered average from stochastic stimulation of squid giant axons. Squid giant axons were stimulated with our stochastic paradigm consisting of model EPSCs and IPSCs [1]. The recording from which this result was taken contained 21 spikes (record duration was 100 sec). All 21 spikes are shown superimposed in the top panel using the maximum amplitude of each spike as a reference point. The underlying current waveforms were similarly aligned (middle panel). The average of these results ±2 SEM is shown in the bottom panel. This result is our candidate optimal stimulus waveform.

An illustration of our experimental results [1] is given in Figure 1, a 100 sec tracing in which 21 action potentials occurred. We determined the features of the input signal that triggered spikes by superimposing all 21 action potentials (Figure 1). The underlying stimulus traces were also superimposed (Figure 1, middle panel; bottom panel, average ±2 SEM). This biphasic waveform is our candidate optimal stimulus shape. It elicited a spike when applied to the same preparation from which the results in Figure 1 were obtained. The root mean square (RMS) current required to elicit a spike with this shape was 40% less than that of a rectangular pulse [1].

**Figure 2:**
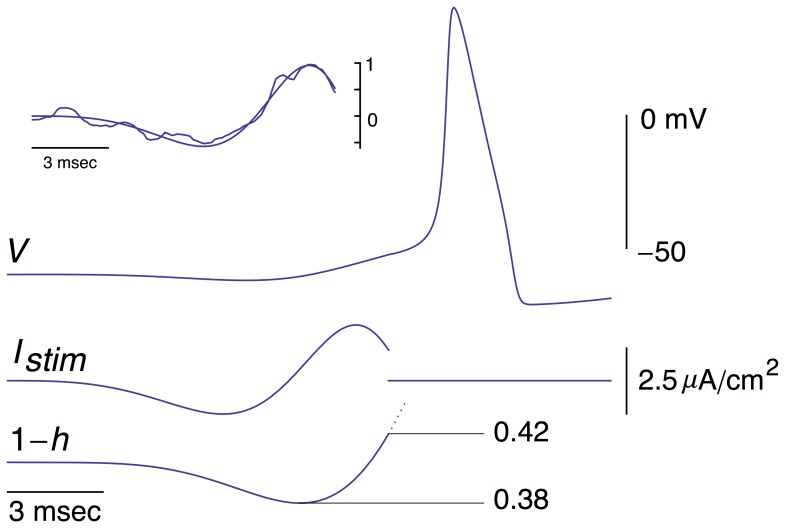
Inset: Another example of an optimal stimulus obtained from a different preparation than the one used in Figure 1. This result is shown superimposed on an *ad hoc* description of this waveform by the expression *f*(*t*) = *a* sin((*bt*)^2^ (exp(*ct*)−1) with *a* = 202 (arbitrary units; this value was chosen so that the maximum amplitude of *f* is 1, *b* = 0.2 msec^−1^, and *c* = 0.00045 msec^−1^. The experimental result has also been scaled so that its maximum value is 1. The response of the Hodgkin and Huxley model to *f*(*t*) is shown, tracing *V*, with *a* = 610 mA/cm^2^. Below that result is the stimulus *f*(*t*) = *I_stim_*, and 1−*h*. The dashed line indicates that the *h* variable continues to change throughout the simulation.

Another example of the optimal stimulus is shown in Figure 2, top left inset, along with a description of this result by the expression given below (Equation 5). The scale was chosen so that the maximum amplitude of the stimulus was unity. Note that the stimulus was truncated at the point for which no additional current was required to elicit a spike. For the purposes of the analysis that follows, an *ad hoc* mathematical function, *f*(*t*), was used with
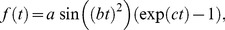
(5)and *a* = 202, *b* = 0.2 msec^−1^, and *c* = 0.00045 msec^−1^. The experimental result in the inset of Figure 2 is shown superimposed upon *f*(*t*).

### Ionic mechanism of response to the optimal stimulus

**Figure 3:**
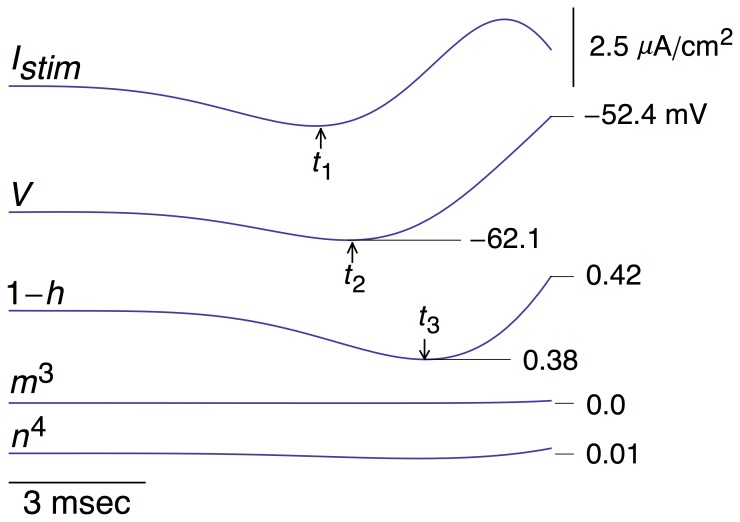
Same result as in **Figure 2** with an emphasis on times of occurrence of the minima in *I_stim_*, *V*, and 1−*h*; *t*_1_, *t*_2_, and *t*_3_, respectively. Relatively little change occurs in *m* ^3^ and *n* ^4^, as indicated in the bottom two tracings. These results are on the same scales as the 1−*h* tracing.

The simulated response of the Hodgkin and Huxley model to the function in Equation 5 was determined with the amplitude of the stimulus, parameter *a*, chosen to be slightly suprathreshold (Figure 2). A slight hyperpolarization occurred prior to the spike, also observed experimentally [1], which produced an *increase* in *h*(*t*), i.e., an increase in availability of *Na* ^+^ channels for activation by the subsequent depolarizing phase of the stimulus. This result is equivalent to a *decrease* in 1−*h*(*t*). The timing of the minimum value prior to the spike of each of the three tracings in Figure 2 is shown in greater detail in Figure 3. The minimum in the membrane potential, *V*, lags behind the minimum of the optimal stimulus. This result, which is attributable to the *RC* time constant of the membrane, would occur if the stimulus were applied to an electrical circuit consisting of a resistor, *R*, in parallel with a capacitor, *C*. Time is required to charge the capacitor, hence the delay in the response of the circuit to the stimulus. The 1−*h*(*t*) variable lags behind a change in *V* due to its time dependence as does *K* ^+^ channel gating (*n* ^4^). In contrast, the *m* gate responds quickly to changes in *V*. Changes in *n* ^4^ and *m* ^3^ are insignificant during the optimal stimulus (bottom two tracings in Figure 3). The level of resting *K* ^+^ channel conductance is small, *n* ^4^ = 0.01. However, that level corresponds to a current of 4.45 µA/cm^2^, which is significant for the subthreshold potential range. (Resting *I_Na_* and *I_L_* are −1.15 and −3.3 µA/cm^2^, respectively). Note that Δ(1−*h*) changes by a relatively small amount during the stimulus, D(1−*h*) = 0.04. This result is to be compared with the change of (1−*h*) that occurs during a rectangular depolarizing pulse, as described below (DISCUSION).

### Relationship of Na^+^ channel inactivation to optimality

**Figure 4:**
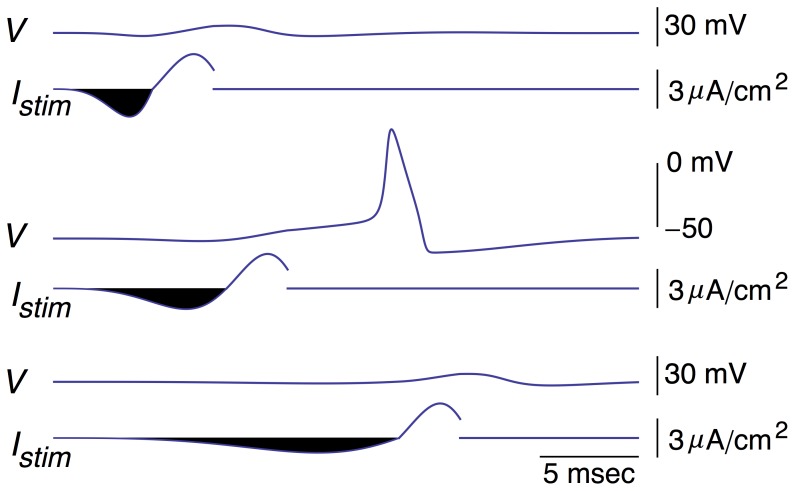
Effect of changing the duration of the hyperpolarizing phase of the biphasic stimulus on firing while keeping the square of the area of this phase constant. Middle panel: *I_stim_* = *f*(*t*) = *a* sin((*bt*)^2^) (exp(*ct*)−1) with *a* = 563 µA/cm^2^, *b* = 0.2 msec^−1^, and c = 0.00045 msec^−1^. These same values of *a*, *b*, and *c* were used for the depolarizing phase of *f*(*t*) in the top and bottom panels, respectively, with the time at the beginning of this phase chosen to match the end of the preceding hyperpolarizing phase. In the top panel, *a* = 1320 µA/cm^2^ and b = 0.35 msec^−1^ for the hyperpolarizing phase (c = 0.00045 msec^−1^ throughout all simulations). In the bottom panel *a* = 201 µA/cm^−2^ and *b* = 0.1 msec^−1^ for the hyperpolarizing phase. These values of *a* were chosen so that the square of the area of the hyperpolarizing phase (shaded black in all 3 panels) was maintained constant as determined using the NIntegrate function in Mathematica.

**Figure 5:**
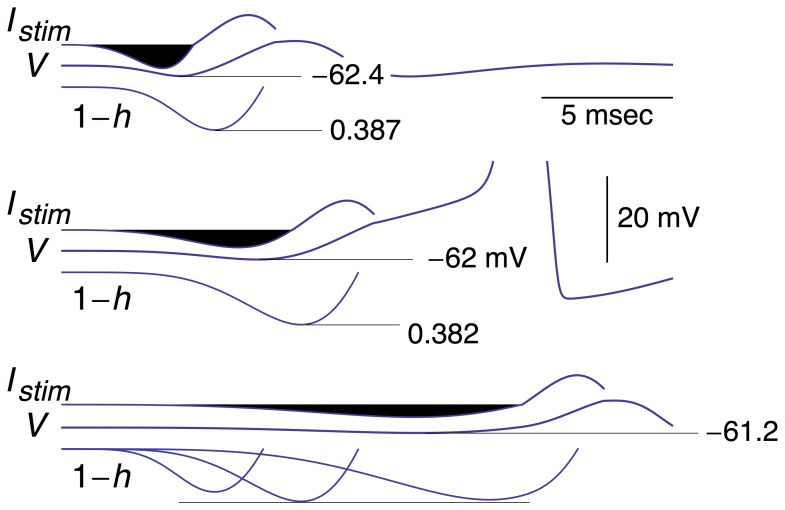
Same results as inFigure 4 with an expanded scale for the voltage axis of the three results and with 1−*h* shown below each simulation. All three 1−*h* results are shown superimposed in the bottom panel. Note that the minimum level of *V* reached during the stimuli is, top to bottom, −62.4, −62, and −61.2 mV, respectively. The minimum level of *h* (removal of *Na* ^+^ channel inactivation) is, top to bottom, 0.387, 0.382, and 0.385, respectively.

We addressed the effect of the timing of the hyperpolarizing phase of the optimal stimulus on the response of the Hodgkin and Huxley model (Figures 4 & 5). This phase is highlighted in black for emphasis. Its kinetics were modified by changing *b* from 0.2 to 0.35 msec^−1^ in the expression for *f*(*t*), and adjusting *a* (Equation 5) so that the square of the shaded area, a measure of energy expenditure, was the same as the square of the shaded area in the middle panel of Figures 4 & 5. (The NIntegrate function of Mathematica was used in this analysis.) The depolarizing phase was kept the same but shifted in time so that it began at the end of the hyperpolarizing phase, i.e., the time at which the anodic portion of the stimulus reaches zero as determined by Equation (5) with *b* = 0.35 msec^−1^. For the cathodic part of the waveform *b* = 0.2 msec^−1^ – the same for the cathodic portion of the stimulus in all the simulations. This stimulus failed to elicit a spike. Similarly, we extended the duration of the hyperpolarizing phase (bottom panels of Figures 4 & 5) by using *b* = 0.1 msec^−1^ and adjusting *a* so that the square of the area of the shaded phase was, again, the same as in the middle panel. This stimulus also failed to elicit a spike. The changes in the 1−*h*[*t*] variable for all three results are illustrated in Figure 5.

**Figure 6:**
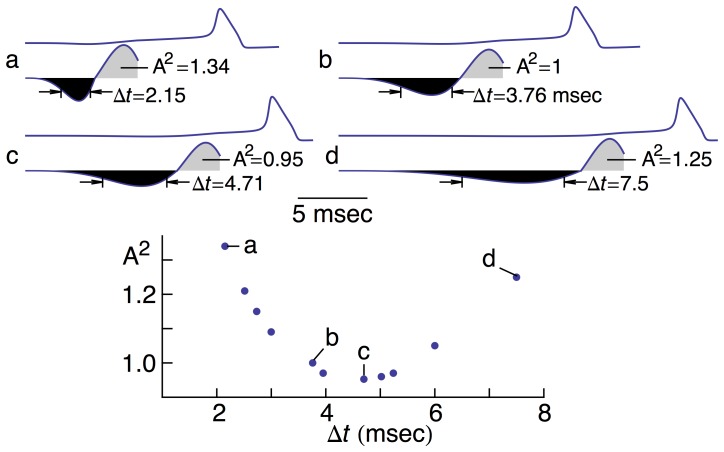
U-shaped dependence of firing on the half-width, Δ*t*, of the hyperpolarizing phase. [The Δ*t* variable is defined as the difference in time between the two points on the hyperpolarizing phase of the stimulus where the amplitude of the curve is one-half the maximum value of the hyperpolarizing current.] Eleven different values of *b* (Equation 5) were used (graph in bottom panel) with *a* in Equation 5 chosen to maintain constancy of value of the square of the area of the phase (shaded black). The depolarizing phase (shaded gray) was adjusted to be slightly suprathreshold as indicated by the simulations in the top panels for **a**–**d**. Panel **b** corresponds to the result in the middle panel of Figures 4 & 5. This is our standard optimal stimulus shape. During the analysis an increase in optimality relative to our standard shape was discovered as indicated by the values of A^2^ in the graph that are less than 1. This parameter – A^2^ – refers to the square of the grey shaded areas in **a**–**d**, a result related to energy expenditure, as noted in the text.

The above results suggest a U-shaped dependence of firing on the parameters of the hyperpolarizing phase of the stimulus, in particular the minimum value of *h* attained during this phase (Figure 5, bottom panel), and its width, Δ*t*, which corresponds to the difference between the times at which the anodic current is one-half of its maximum, or peak, value. This value occurs both on the rising and declining phases of the curves that border the black shaded areas in Figure 6. We adjusted the amplitudes of the depolarizing phases of the stimulus (light shaded areas in Figure 6) until they were slightly suprathreshold. We found a slight *increase* in optimality (∼5% reduction in the size of the square of the area) when the duration of the hyperpolarizing phase was increased by ∼25%. We note again that throughout this analysis the square of the area of the hyperpolarizing phase of the stimulus, a measure of energy expenditure, was maintained constant. In our recent work the time constants of IPSCs and EPSCs were the same. Results from the literature suggest that the decay times of IPSCs are longer than those of EPSCs [16]. The analysis in Figure 6 is serendipitously consistent with these results and a testable prediction for squid as well as other preparations.

A surprising result of our analysis (not shown) is the increase in amplitude of the depolarizing phase of the stimulus (light shaded areas in Figure 6) required to elicit a spike in the absence of the hyperpolarizing phase. An increase of 1.7 (using the result in panel **b** of Figure 6 as baseline) was needed, which corresponds to an increase of 2.89 in the square of the area (A^2^ in Figure 6). That is, neither side of the U-shaped curve (bottom panel of Figure 6) increases without limit. Rather, each side approaches 2.89. This result is obtained in the limit of long duration hyperpolarizing currents preceding the depolarizing phase, Δ*t*→∞, or for very brief durations with a delta function as the limiting shape of the hyperpolarizing phase, Δ*t*→0. Our conclusion from these results is that the hyperpolarizing phase, regardless of its duration, significantly reduces the amount of current in the subsequent depolarizing phase required to elicit a spike and that a fairly broad range of durations in the vicinity of the minimum of the U-shaped curve (Figure 6) provides an optimal, or near-optimal, stimulus shape.

## Discussion

**Figure 7:**
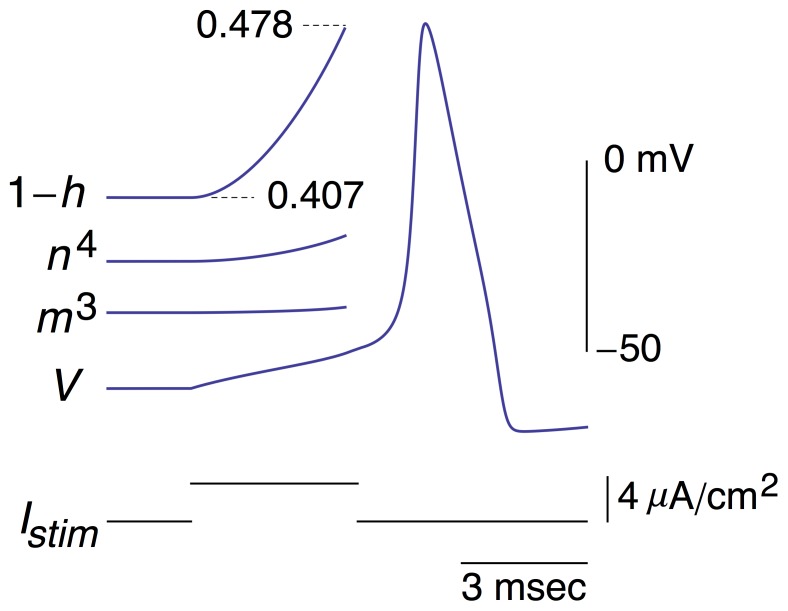
Ionic mechanism underlying response of the Hodgkin and Huxley model to a rectangular depolarizing current pulse. The 1−*h*, *n* ^4^, and *m* ^3^ results correspond to the changes in these parameters from the beginning of the stimulus (*I_stim_*) until a point near threshold similar to that of the optimal stimulus in Figures 2 and 3. The scales for these results are the same as in Figure 3. Note in particular changes that occur in both *n* ^4^ and 1−*h*, in contrast the results in Figure 3, as well as the significantly larger change in 1−*h* relative to the results in Figures 2 and 3.

In our recent study [1], we demonstrated both theoretically using the Hodgkin and Huxley model, and experimentally, using stimulation of the squid giant axon preparation, an optimal stimulus shape for eliciting a spike from the axon. These results were context dependent. That is, they depended upon the input to the axon. In these experiments the input consisted of: 1) a mixture of EPSCs and IPSCs each having a decay time constant of 1 msec; 2) only IPSCs each having a decay time constant of 1 msec or; 3) a mixture of EPSCs and IPSCs each having a decay time constant of 20 msec. The spike-triggered average for each had a marked hyperpolarizing phase that, as the analysis here has demonstrated, removes some of the resting *Na* ^+^ channel inactivation [1]. The three waveforms represent local optima. Of the three the first requires the lowest RMS current amplitude to elicit a spike. Consequently, we focused on that result in this report. As noted, the underlying mechanism is a change in *Na* ^+^ channel inactivation, the *h* gate, during the stimulus waveform. This is novel analysis that does not appear to have been described in previous reports. The results in Figure 3 provide a clue as to the fundamental reason for optimality of the waveform described above [RESULTS}. The only change in gating variables is in *h*. Insignificant changes occur in activation gating for either *Na* ^+^ or *K* ^+^ channels. A mechanism of this type would seem to be more efficient than one in which changes occur in more than a single variable. By contrast changes in both *h* and *n^4^*, occur during a slightly suprathreshold rectangular depolarizing pulse, i.e., a cathodic pulse – the traditional way of eliciting a spike (Figure 7). Furthermore, the *h* parameter is changed during a rectangular pulse by a factor that is almost twice as great (Figure 7) as the changes in *h* that occur during the optimal stimulus (Figures 2 and 3). These results for *h* and *n* ^4^ may underlie the 40% reduction in RMS current required to elicit a spike with the optimal waveform compared to a rectangular depolarizing pulse.

As described in this report, the hyperpolarizing phase of the optimal stimulus is a crucial component of the waveform. Hyperpolarizing current is well known to enhance neuronal excitability [13]. At one end of this spectrum is anodal break excitation, a phenomenon observed in many neurons. For example Lacunosum-Moleculare [L-M] interneurons in the CA1 region of the hippocampus exhibit this effect [17]. The excitatory mechanism in these cells is a low threshold, transient *Ca* ^2+^ current [18]. Consequently, a hyperpolarizing current pulse applied to these cells removes resting *Ca* ^2+^ channel inactivation and resting *K* ^+^ channel activation.

The difference between removal of resting *Na* ^+^ channel inactivation and resting *Ca* ^2+^ channel inactivation is not significant in a global context in the determination of stimulus optimality. We predict our experimental protocol applied to L-M interneurons with all synaptic inputs to those cells blocked would yield a biphasic waveform similar to our results from squid axons. That stimulus could be used to trigger an IPSP in the post-synaptic CA1 pyramidal neuron, the response of those cells to L-M interneuron stimulation [19]. In this way our concepts of optimality could, in principle, be tested in a circuit context.

CA1 pyramidal neurons do not appear to exhibit anodal break excitation based on results in the literature in contrast to pyramidal neurons in other regions of the brain that do exhibit this effect [13]. This result, i.e., the lack of anodal break excitation, can be mimicked in the Hodgkin and Huxley model (METHODS) by the use of steady hyperpolarizing current that is sufficient to bring the rest (reference) potential below the activation range of the delayed rectifier. For example, a steady hyperpolarizing current of 4.5 µA/cm^2^ gives a reference potential of −65 mV. For these conditions the model does not exhibit anodal break excitation (simulations not shown).

An analysis such as ours on subthreshold ionic mechanisms underlying optimal responses of squid giant axons would appear to apply to many neurons. For example, sodium ion current along with a low-threshold potassium ion current both contribute to the shape of spike-triggered averages in auditory neurons [20]. Our approach offers considerably flexibility in the analysis of optimality for this and other preparations. The parameters of our model EPSCs can be changed independently from those of the IPSCs and conversely, including complete elimination of either one or the other. This flexibility, which is not available in the standard “white noise” approach, can be used in future studies concerning optimality in specific neuronal contexts.

### Biphasic stimuli and optimality

Biphasic stimuli have been used in neuroscience in a variety of applications both in cellular neuroscience and in the clinic. The original motivation for their use with chronically implanted electrodes in patients experiencing neurological disorders was to achieve charge neutralization [21]. For example, medically refractory movement disorders associated with Parkinson's disease (PD) are currently treated using deep brain stimulation – DBS [3], [4], [21]. Given that DBS electrodes are chronically implanted in the brains of patients with PD and that electrical pulses are continuously applied by means of a battery to achieve therapeutic benefit, charge balance is a necessary requirement of this procedure [21]. Consequently, biphasic pulses are used containing anodal and cathodal portions in which the areas of the two phases are matched [21]. Optimization of the relative timing and shape of the two phases for efficiency of stimulation may help to reduce the frequency of battery replacement surgery [3]. For example, recent experiments concerning stimulus optimization of peripheral nerves in adult cats have shown that more efficient stimulation occurs when the anodic portion of the pulse precedes the cathodic part rather than the reverse order [3], [4], a result consistent with the analysis in this report. Additionally, an increase in optimality was obtained when a smoothly varying Gaussian shape was used for the cathodic portion of the stimulus [3]. Our results suggest that a smoothly varying waveform for the anodic portion as well might further increase efficiency of stimulation. The target of stimulation in experiments on peripheral nerves is axons. Similarly, both DBS and cortical stimulation are also thought to occur via axons [2], [22], [23]. DBS involves extracellular stimulation of neurons. Our analysis is concerned with intracellular stimulation. The two approaches are related. For example, asymmetrical charge-balanced biphasic stimuli consisting of a long-duration low-amplitude cathodic prepulse followed by a short-duration high-amplitude anodic stimulus phase have been shown to selectively activate targeted neuronal populations in the brain when applied extracellulary [24]. We have found similar stimuli to be optimally effective when applied intracellulary. Moreover, the mathematical relationship between extra- and intracellular stimulation is straightforward for axons [25]. Mammalian nonmyelinated axons have relatively few ionic current components, *I_Na_*, *I_K_*, and *I_L_* in particular, and propagation of spikes along those axons can be simulated using those components in a manner similar to that of the original Hodgkin and Huxley analysis of squid giant axons [26].

Optimality with biphasic stimuli has been considered in other contexts. For example, a brief well-timed inhibitory influence can enhance a subthreshold excitatory input to brain stem auditory neurons thereby facilitating spiking in those cells, a process termed postinhibitory facilitation [27]. These results and ours are two examples of an enhancement of an excitatory effect by a preceding inhibitory effect, a mechanism at the cellular level that may be widespread throughout the nervous system.
